# Nitrogen Use Efficiency in Oil Palm Seedlings: Unraveling the Untapped Potential of Elevated External Ammonium Supply

**DOI:** 10.3390/plants12152819

**Published:** 2023-07-30

**Authors:** Marlon De la Peña, Rodrigo Ruiz-Romero, Hernán Mauricio Romero

**Affiliations:** 1 Oil Palm Biology and Breeding Research Program, Colombian Oil Palm Research Center—Cenipalma, Bogotá 11121, Colombia; malondlp@gmail.com (M.D.l.P.); rruiz@cenipalma.org (R.R.-R.); 2 Department of Biology, Universidad Nacional de Colombia, Bogotá 11132, Colombia

**Keywords:** nitrogen use efficiency, growth, metabolites, ammonium tolerance

## Abstract

Ammonium (NH_4_^+^) is an essential nitrogen source for plants, but excessive exposure can trigger stress responses that vary among and within different plant species. This study investigated the phenotypic variations in response to ammonium nutrition in five oil palm genotypes seedlings. Nitrate nutrition was used as a reference for a non-stressful condition, and three different nitrogen concentrations (5, 10, and 15 mM) were examined. Control groups without external nitrogen application were included for each genotype. Several parameters were analyzed, including plant growth, root length, gas exchange, fluorescence, chlorophyll, reducing sugars, amino acids, proteins, and nitrogen uptake. The results revealed a significant genotype effect, particularly between the interspecific OxG hybrid and the *Elaeis guinensis* genotypes. Ammonium nutrition increased shoot growth in all genotypes compared to nitrate nutrition. Additionally, there was a trend towards increased primary root length, amino acids, proteins, and nitrogen uptake under ammonium supply. These findings are promising, particularly considering the recommendation to use ammonium with inhibitors for environmental sustainability.

## 1. Introduction

Colombia is the leading producer of oil palm (*Elaeis guineensis* Jacq.) in Latin America and the fourth-largest producer in the world, with 559,582 hectares cultivated. Oil palm is essential for food production and is a crucial industrial raw material, making it a significant contributor to the economy of several countries. To meet the nutritional needs of this crop, mature palms typically receive 100–200 kg of nitrogen per hectare per year. These fertilizer applications, which account for a substantial portion (46–85%) of production costs [1], can be strategically managed to improve profitability and promote sustainability.

Nitrate and ammonium are the primary nitrogen sources used in these applications. However, their indiscriminate use can lead to ecological issues. For example, nitrate fertilizers can produce nitrous oxide (N_2_O), a potent greenhouse gas with a global warming potential 298 times higher than carbon dioxide [2]. Moreover, nitrate exhibits high mobility in the soil, posing a risk of groundwater contamination and causing eutrophication in water bodies. On the other hand, ammonium can bind to soil particles, making it less prone to leaching than nitrate. Nevertheless, it can still be converted into nitrate within the soil or be released as ammonia (NH_3_) into the atmosphere, with approximately 1% of ammonia indirectly converting to N_2_O [2].

The Intergovernmental Panel on Climate Change recommends using urease and nitrification inhibitors as a potential solution to mitigate NH_3_ and N_2_O emissions in agriculture [3]. These inhibitors offer a notable advantage by extending the presence of ammonium in the soil, thereby facilitating its uptake by plants. Furthermore, urease and nitrification inhibitors have been shown to reduce NH_3_ and N_2_O emissions [4]. In this regard, ammonium nutrition emerges as a promising strategy to enhance nitrogen use efficiency (NUE), which essentially refers to the amount of nitrogen effectively utilized and recovered in the crop yield. 

Supplying ammonium as the sole nitrogen source can induce stress at lower concentrations compared to nitrate, and this response can vary significantly, even within the same species [5]. Conversely, nitrate is generally the primary nitrogen source for plants, as it is the most abundant form of nitrogen in agricultural soils. Many crops have been bred and selected under nitrate conditions, potentially resulting in the loss of adaptive mechanisms for ammonium nutrition. Nevertheless, there are exceptions to this trend, particularly in crops that thrive in acidic soils like rice, where nitrification is limited, leading to a higher prevalence of ammonium as a nitrogen source. Therefore, it is essential to conduct plant-specific studies for more precise information on the exposure at which ammonium becomes stressful for specific plant species.

In oil palm, a previous study examined ammonium as the sole nitrogen source for young oil palm trees (7 months old) [6]. The study found similar performance between ammonium and nitrate nutrition. However, this experiment was conducted in neutral soil, which introduces the possibility of ammonium being converted to nitrate by nitrification bacteria over time. Therefore, the objective of our study was to assess the performance, physiological parameters, and specific metabolites involved in the response of oil palm seedlings to ammonium nutrition compared to nitrate nutrition. We utilized different nitrogen concentrations in an inert substrate that limited nitrification. We focused on five economically significant oil palm genotypes that display phenotypic diversity. This research will provide valuable insights into improving nitrogen use efficiency (NUE) and promoting sustainable and environmentally responsible management practices in oil palm cultivation.

## 2. Results

### 2.1. Multivariate Analysis

**Figure 1:**
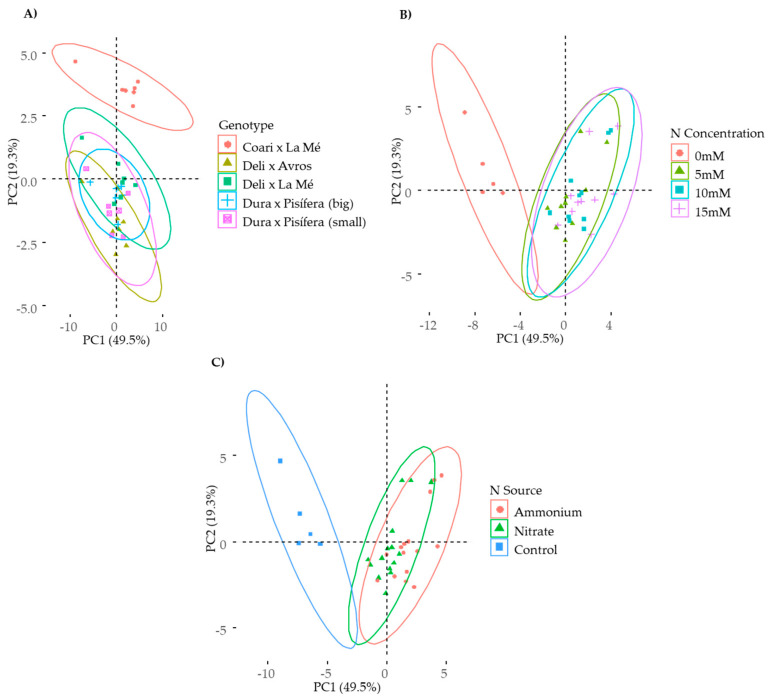
Principal component analysis (PCA) for five oil palm genotypes under ammonium and nitrate nutrition at 5, 10, and 15 mM and a control condition (0 N). Variables considered: dry shoot weight, dry root weight, dry root-to-shoot ratio, primary root (PR) length, lateral root (LR) length, net photosynthetic rate (A_n_), stomatic conductance (g_s_), intercellular CO_2_ (C_i_), transpiration rate (E), maximum quantum yield of PSII (F_v_/F_m_), electron transport rate (ETR), quantum yield of photosystem II (PhiPS2), non-photochemical quenching (NPQ), chlorophyll content (Chl), leaf reducing sugars, root reducing sugars, leaf amino acids, root amino acids, leaf proteins, root proteins, shoot N uptake, and root N uptake. Sample score of the individuals for the first (PC1) and second (PC2) principal components for (**A**) genotype, (**B**) nitrogen (N) concentration, (**C**) nitrogen source, and control.

Twenty-two parameters were subjected to principal component analysis (PCA). The results revealed that the first two components of the PCA accounted for a significant portion of the variance (approximately 69%). By employing different groupings, the PCA discriminated between *E. guineensis* genotypes and the interspecific OxG hybrid (Figure 1A), between the control plants and those subjected to varying levels of N concentrations (Figure 1B), and between different N sources (Figure 1C). The interspecific OxG hybrid CxLM was vertically spread at the top, while the *E. guineensis* cultivars were predominantly distributed at the bottom (Figure 1A). Furthermore, the nitrogen-supplied plants were positioned along the horizontal plane towards the right, while the control plants were towards the left (Figure 1B,C).

**Figure 2:**
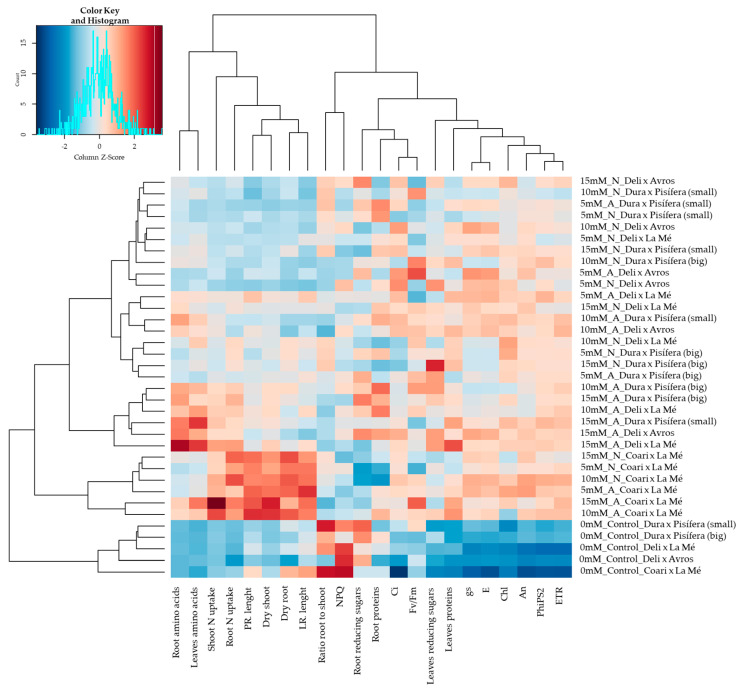
Hierarchical clustering analysis (HCA) of five oil palm seedling genotypes under ammonium (A) and nitrate (N) nutrition conditions, including 5, 10, and 15 mM concentrations and a control (0 N) condition. Variables considered: dry shoot weight, dry root weight, dry root-to-shoot ratio, primary root (PR) length, lateral root (LR) length, net photosynthetic rate (A_n_), stomatic conductance (g_s_), intercellular CO_2_ (C_i_), transpiration rate (E), maximum quantum yield of PSII (F_v_/F_m_), electron transport rate (ETR), quantum yield of photosystem II (PhiPS2), non-photochemical quenching (NPQ), chlorophyll content (Chl), leaf reducing sugars, root reducing sugars, leaf amino acids, root amino acids, leaf proteins, root proteins, shoot N uptake, and root N uptake.

The hierarchical clustering analysis (HCA) revealed the formation of a distinctive cluster that appeared to comprise all the control treatments (Figure 2) exclusively. This cluster exhibited a positive correlation with a smaller cluster of variables, including NPQ (non-photochemical quenching) and the root-to-shoot ratio. Conversely, it displayed a negative association with a cluster of variables encompassing leaf-reducing sugars, leaf proteins, stomatal conductance (g_s_), transpiration rate (E), chlorophyll (Chl), net photosynthetic rate (A_n_), quantum yield of photosystem II (PhiPS2), and electron transport rate (ETR). Another distinctive cluster was observed, which exclusively comprised the interspecific OxG hybrid CxLM. This cluster displayed a positive relationship with variables such as N uptake and dry weight in shoots and roots as well as primary and lateral root length.

### 2.2. Growth and Development

**Figure 3:**
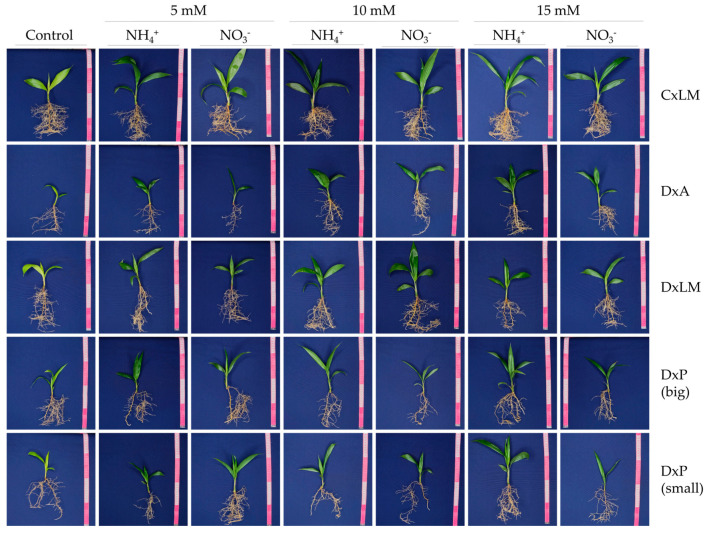
Plant growth of interspecific OxG hybrids (CxLM) and *E. guineensis* DxA, DxL, DxP (big), and DxP (small)) genotypes under a control condition (0 N) and different N sources (ammonium or nitrate) at three levels (5, 10, and 15 mM).

Nitrogen nutrition in the form of ammonium and nitrate impacts plants at the morphological, physiological, and metabolic level. Although high ammonium concentrations can harm plants, no visible signs of toxicity were observed in 14-week-old seedlings, even after continuous exposure for seven weeks. However, leaf chlorosis was observed in the control group (0 N) due to the prolonged absence of nitrogen (Figure 3). 

**Figure 4:**
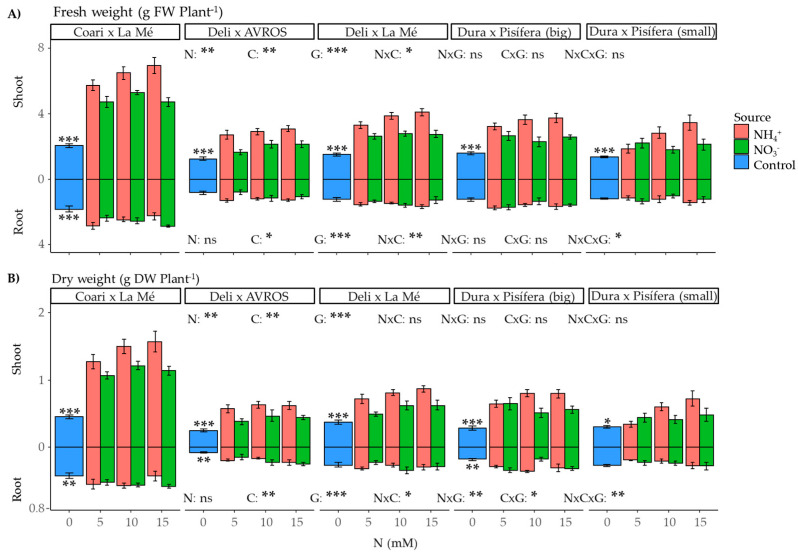
Average (**A**) fresh and (**B**) dry shoot and root weight for each genotype grown under each nitrogen source (ammonium or nitrate) for three concentrations (5, 10, and 15 mM) and a control treatment (0 N). Statistical significance levels for the main effects and interactions among nitrogen source (N), nitrogen concentration (C), and genotype (G) are denoted by asterisks (* 0.05, ** 0.01, and *** < 0.001), and non-significant results are labeled as (ns). Significant differences in the orthogonal contrast between nitrogen treatments and the control are indicated with an asterisk above the control (blue) bar. Error bars represent standard errors (*n* = 5).

We evaluated fresh and dry weights to assess oil palm performance under different nitrogen conditions. The nitrogen source (*p* < 0.01), concentration (*p* < 0.01), and genotype (*p* < 0.001) significantly influenced shoot fresh and dry weights (Figure 4A). Furthermore, the interaction between nitrogen source and concentration (*p* < 0.05) revealed that the difference in shoot fresh weight production between ammonium and nitrate sources became more prominent with increasing concentrations. The statistical analysis comparing the control groups with the nitrogen-treated groups revealed a notable difference (*p* < 0.05) in dry weight for DxP (small) and a highly significant difference (*p* < 0.001) for the remaining contrasts of shoot weight.

In the root, the nitrogen concentration (*p* < 0.05 for fresh weight; *p* < 0.001 for dry weight) and genotype (*p* < 0.001) were identified as influential factors (Figure 4B). Additionally, significant interactions were observed between nitrogen source and concentration (*p* < 0.01 for fresh weight; *p* < 0.05 for dry weight), as well as between nitrogen source, concentration, and genotype (*p* < 0.05 for fresh weight; *p* < 0.01 for dry weight). The interactions between nitrogen source and genotype (*p* < 0.01) and between nitrogen concentration and genotype (*p* < 0.05) were also found to be significant for dry weight (*p* < 0.01). Comparing the control group with nitrogen-treated groups revealed highly significant contrasts for CxLM in fresh weight (*p* < 0.001) and for CxLM, DxA, and DxP (big) in dry weight (*p* < 0.01).

**Figure 5:**
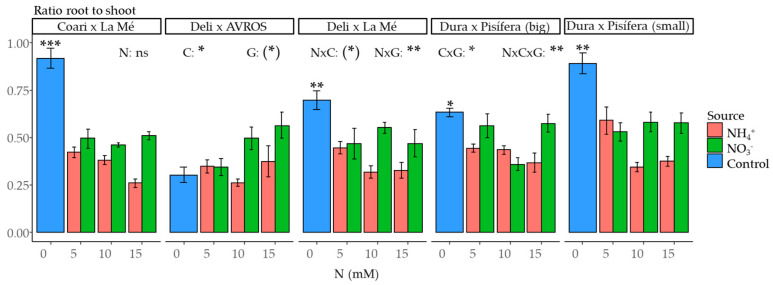
Average root-to-shoot dry weight ratio for each genotype grown under each nitrogen source (ammonium or nitrate) for three concentrations (5, 10, and 15 mM) and a control treatment (0 N). Statistical significance levels for the main effects and interactions among nitrogen source (N), nitrogen concentration (C), and genotype (G) are denoted by asterisks ((*) 0.1, * 0.05, ** 0.01, and *** < 0.001), and non-significant results are labeled as (ns). Significant differences in the orthogonal contrast between nitrogen treatments and the control are indicated with asterisks above the control (blue) bar. Error bars represent standard errors (*n* = 5).

The root-to-shoot ratio, determined based on dry weight, was significantly impacted by nitrogen concentration (*p* < 0.05) and the interactions of nitrogen source and genotype (*p* < 0.01), nitrogen concentration and genotype (*p* < 0.05), and the triple interaction of nitrogen source, concentration, and genotype (*p* < 0.01) (Figure 5). For the genotype DxA, the contrast between the control and nitrogen-treated groups did not show statistical significance. However, for the remaining genotypes, there was a significant difference (*p* < 0.001 for CxLM, *p* < 0.01 for DxLM and DxP (small), and *p* < 0.05 for DxP (big)).

**Figure 6:**
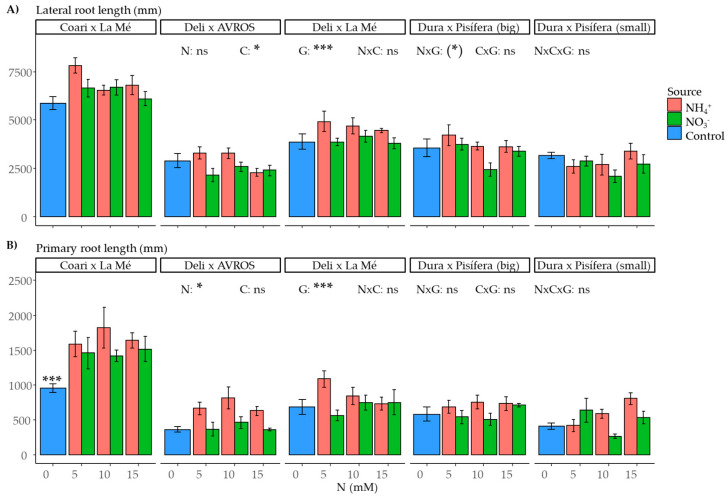
Average (**A**) lateral and (**B**) primary root length for each genotype grown under each nitrogen source (ammonium or nitrate) for three concentrations (5, 10, and 15 mM) and a control treatment (0 N). Statistical significance levels for the main effects and interactions among nitrogen source (N), nitrogen concentration (C), and genotype (G) are denoted by asterisks ((*) 0.1, * 0.05, and *** < 0.001), and non-significant results are labeled as (ns). Significant differences in the orthogonal contrast between nitrogen treatments and the control are indicated with an asterisk above the control (blue) bar. Error bars represent standard errors (*n* = 5).

The length of roots is crucial in facilitating nutrient absorption from the soil. In this study, the genotype had a significant influence (*p* < 0.001) on lateral root (LR) and primary root (PR) lengths (Figure 6A,B). Furthermore, LR length was affected by nitrogen concentration (*p* < 0.05), while PR length was influenced by the nitrogen source (*p* < 0.05). However, when comparing the control group to the nitrogen-treated groups, we found that the contrast was significant (*p* < 0.001) only for the PR length of CxLM. This indicates that the absence of nitrogen severely affected the primary root length of this genotype.

### 2.3. Leaf Gas Exchange and Fluorescence Parameters

**Figure 7:**
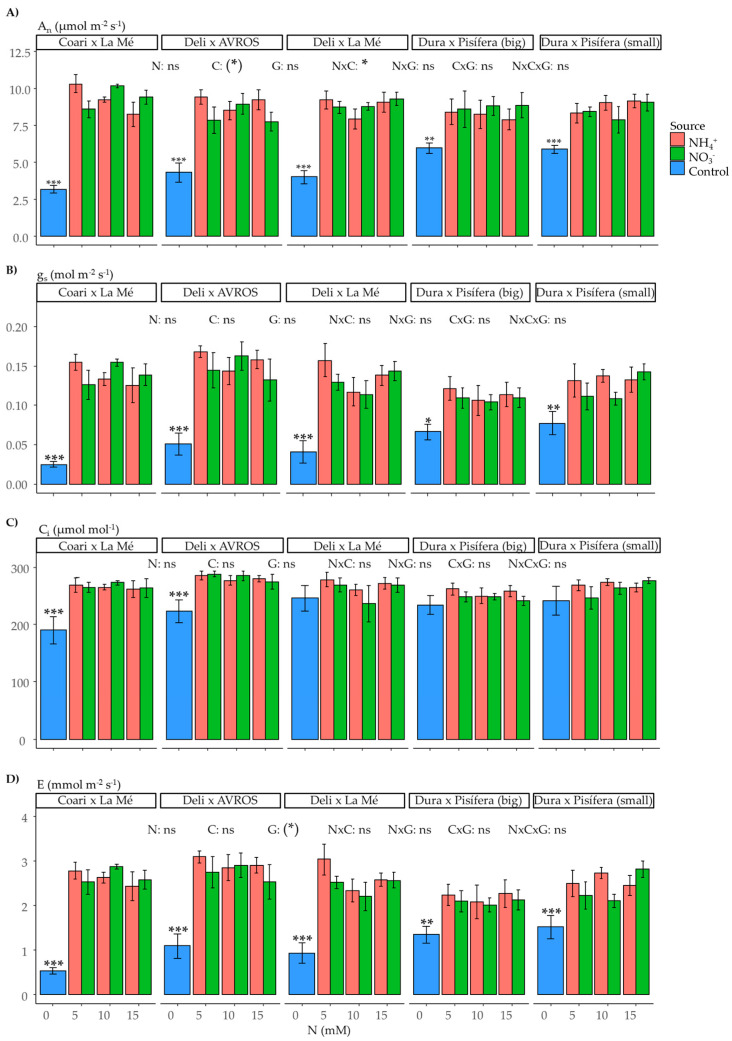
Average values of gas exchange parameters: (**A**) net photosynthetic rate (A_n_), (**B**) stomatic conductance (g_s_), (**C**) intercellular CO_2_, and (**D**) transpiration rate for each genotype grown under each nitrogen source (ammonium or nitrate) for three concentrations (5, 10, and 15 mM) and a control treatment (0 N). Statistical significance levels for the main effects and interactions among nitrogen source (N), nitrogen concentration (C), and genotype (G) are denoted by asterisks ((*) 0.1, * 0.05, ** 0.01, and *** < 0.001), and non-significant results are labeled as (ns). Significant differences in the orthogonal contrast between nitrogen treatments and the control are indicated with an asterisk above the control (blue) bar. Error bars represent standard errors (*n* = 5).

**Figure 8:**
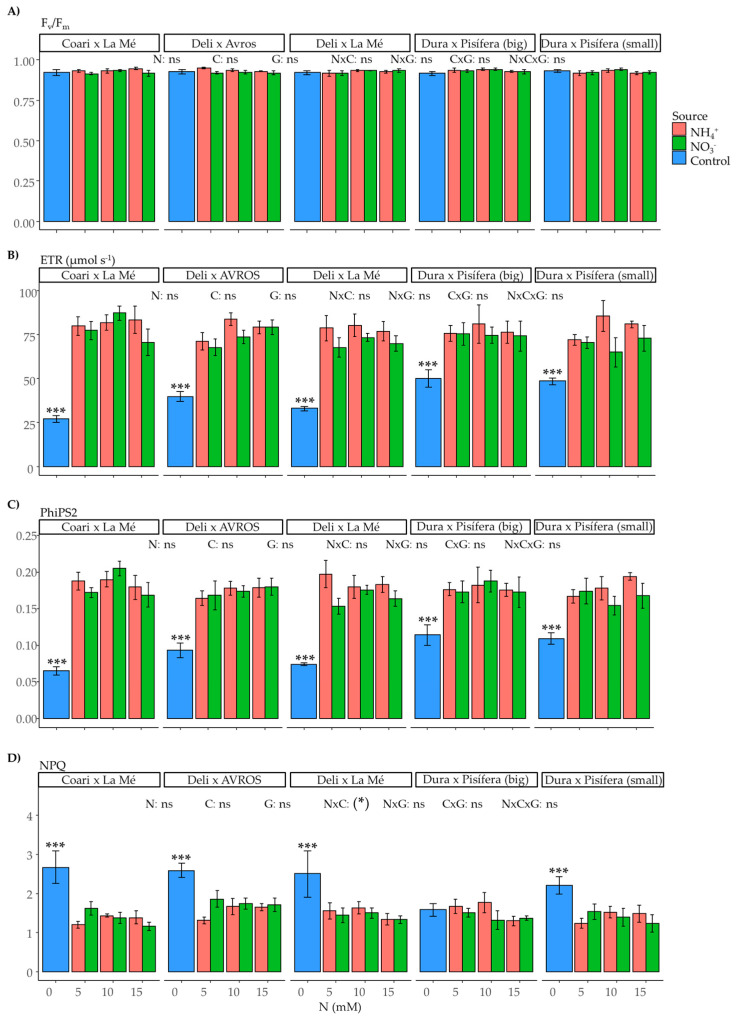
Average values of fluorescence parameters: (**A**) maximum quantum yield of PSII (F_v_/F_m_), (**B**) electron transport rate (ETR), (**C**) quantum yield of photosystem II (PhiPS2), and (**D**) non-photochemical quenching (NPQ) for each genotype grown under each nitrogen source (ammonium or nitrate) for three concentrations (5, 10, and 15 mM) and a control treatment (0 N). Statistical significance levels for the main effects and interactions among nitrogen source (N), nitrogen concentration (C), and genotype (G) are denoted by asterisks (*** < 0.001), and non-significant results are labeled as (ns). Significant differences in the orthogonal contrast between nitrogen treatments and the control are indicated with an asterisk above the control (blue) bar. Error bars represent standard errors (*n* = 5).

Nitrogen is a vital nutrient for supporting photosynthesis and fluorescence. In our study, we observed a slight but significant impact of the interaction between nitrogen source and concentration on the net photosynthetic rate (A_n_) (*p* < 0.05) (Figure 7A). However, no significant effects were observed in the other measured parameters (Figure 7B–D and Figure 8A–D). Furthermore, when comparing the control group to the nitrogen-treated groups, we found significant effects in most of the measurements of photosynthesis and fluorescence in the control groups of each genotype, except for intercellular CO_2_ (C_i_) (not significant for DxLM, DxP (big), and DxP (small)), maximum quantum yield of PSII (Fv/Fm) (not significant for any of the genotypes), and non-photochemical quenching (NPQ) (not significant for DxP (big)).

### 2.4. Metabolite Content

**Figure 9:**
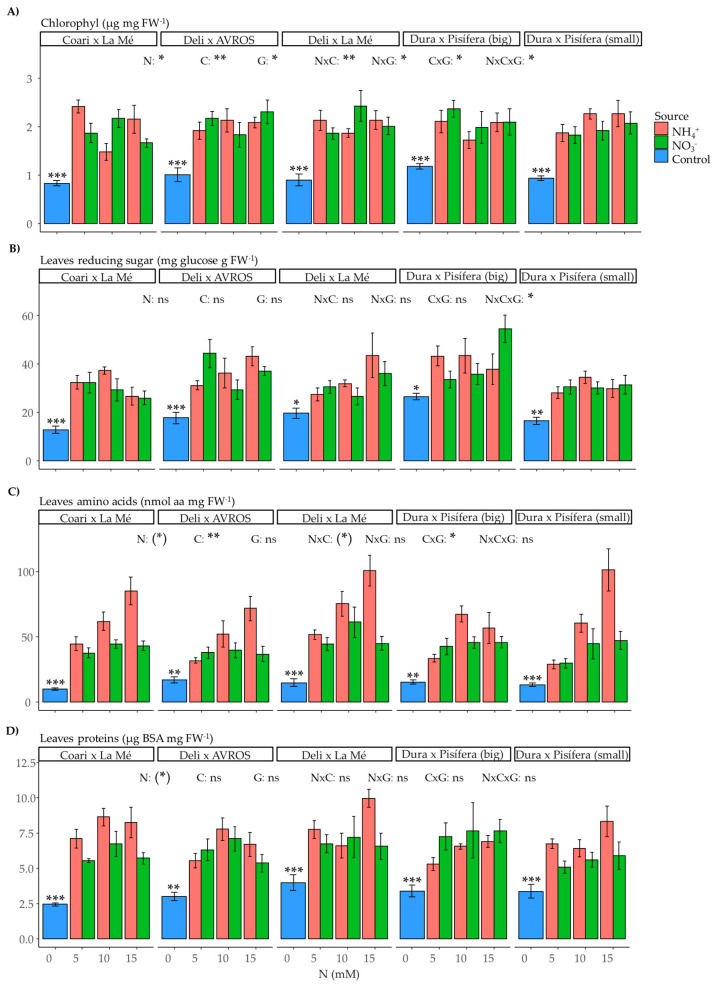
Average values of leaf metabolites: (**A**) chlorophyll, (**B**) reducing sugars, (**C**) amino acids, and (**D**) proteins for each genotype grown under each nitrogen source (ammonium or nitrate) for three concentrations (5, 10, and 15 mM) and a control treatment (0 N). Statistical significance levels for the main effects and interactions among nitrogen source (N), nitrogen concentration (C), and genotype (G) are denoted by asterisks ((*) 0.1, * 0.05, ** 0.01, and *** < 0.001), and non-significant results are labeled as (ns). Significant differences in the orthogonal contrast between nitrogen treatments and the control are indicated with an asterisk above the control (blue) bar. Error bars represent standard errors (*n* = 3–5).

Nitrate in plant cells activates pathways that use energy to convert N from valence +5 to organic compounds of valence −3, while NH_4_^+^ does not need to undergo this process as N is already reduced to valence −3. However, NH_4_^+^ can be toxic to plants at high concentrations, so plants have metabolic mechanisms to avoid this toxicity. In this study, the chlorophyll content was significantly influenced by the nitrogen source (*p* < 0.05), nitrogen concentration (*p* < 0.01), and genotype (*p* < 0.05), as well as the double interactions between nitrogen source and concentration (*p* < 0.01), nitrogen source and genotype (*p* < 0.05), and nitrogen concentration and genotype (*p* < 0.05) and the triple interaction of nitrogen source, concentration, and genotype (*p* < 0.05) (Figure 9A). The levels of reducing sugars in leaves were also affected by the triple interaction (*p* < 0.05) (Figure 9B). Furthermore, the leaf amino acid content was influenced by the nitrogen concentration (*p* < 0.01) and the interaction between nitrogen concentration and genotype (*p* < 0.05) (Figure 9C). However, the leaf protein content did not significantly impact any factors examined (Figure 9D). Interestingly, when comparing the control and nitrogen-treated groups, significant differences were observed in all the metabolites measured in leaves.

**Figure 10:**
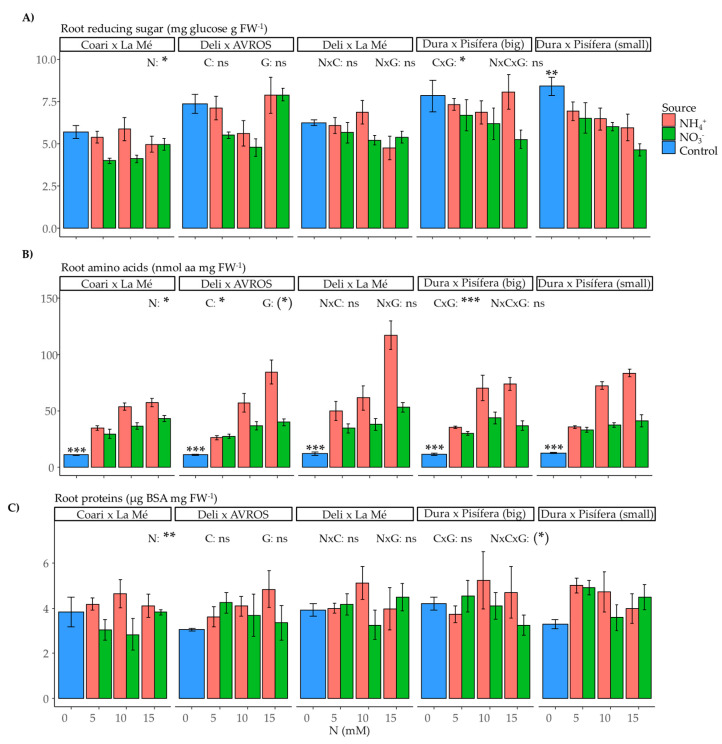
Average values of root metabolites: (**A**) reducing sugars, (**B**) amino acids, and (**C**) proteins for each genotype grown under each nitrogen source (ammonium or nitrate) for three concentrations (5, 10, and 15 mM) and a control treatment (0 N). Statistical significance levels for the main effects and interactions among nitrogen source (N), nitrogen concentration (C), and genotype (G) are denoted by asterisks ((*) 0.1, * 0.05, ** 0.01, and *** < 0.001), and non-significant results are labeled as (ns). Significant differences in the orthogonal contrast between nitrogen treatments and the control are indicated with an asterisk above the control (blue) bar. Error bars represent standard errors (*n* = 3–5).

In the roots, the content of reducing sugars exhibited significant effects as influenced by the nitrogen source (*p* < 0.05) and the interaction between nitrogen concentration and genotype (*p* < 0.05) (Figure 10A). Similarly, the amino acid content in the roots was significantly influenced by the nitrogen source (*p* < 0.05) and concentration (*p* < 0.05) as well as the interaction between nitrogen concentration and genotype (*p* < 0.001) (Figure 10B). On average, ammonium resulted in higher amino acid content compared to nitrate. Moreover, root protein content was significantly affected by the nitrogen source (*p* < 0.01) (Figure 10C), indicating that, on average, ammonium led to higher protein content than nitrate. Regarding the comparison of the control group and the nitrogen treatments, significant differences were observed for root-reducing sugars in DxP (small) (*p* < 0.01), as well as for root amino acid content in all genotypes (*p* < 0.001), but not for root protein content.

### 2.5. Nitrogen Uptake 

**Figure 11:**
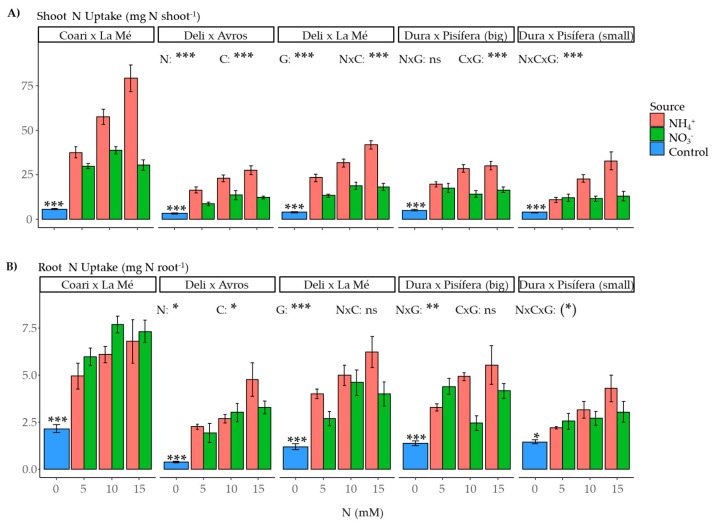
Average nitrogen content for each genotype grown under each nitrogen source (ammonium or nitrate) for three concentrations (5, 10, and 15 mM) and a control treatment (0 N) in (**A**) shoots and (**B**) roots. Statistical significance levels for the main effects and interactions among nitrogen source (N), nitrogen concentration (C), and genotype (G) are denoted by asterisks ((*) 0.1, * 0.05, ** 0.01, and *** < 0.001), and non-significant results are labeled as (ns). Significant differences in the orthogonal contrast between nitrogen treatments and the control are indicated with an asterisk above the control (blue) bar. Error bars represent standard errors (*n* = 5).

The effect of the nitrogen source on N uptake in the shoots was found to be significant, with N uptake being influenced by the nitrogen source (*p* < 0.001), concentration (*p* < 0.001), genotype (*p* < 0.001), and the interactions between nitrogen source and concentration (*p* < 0.001) and nitrogen concentration and genotype (*p* < 0.001) as well as the triple interaction of nitrogen source, concentration, and genotype (*p* < 0.001) (Figure 11A). Similarly, root N uptake was affected by the nitrogen source (*p* < 0.05), concentration (*p* < 0.05), genotype (*p* < 0.001), and the interaction between nitrogen source and genotype (*p* < 0.01) (Figure 11B). On average, N uptake is higher with ammonium than nitrate nutrition. Additionally, the difference between ammonium and nitrate nutrition tends to increase as the concentration of N increases. Finally, when comparing the control and nitrogen-treated groups, the contrasts were significant for both shoots and roots.

## 3. Discussion

Previous studies have provided valuable insights into the response of plant species to different concentrations of ammonium, demonstrating significant variation in their tolerance and sensitivity. For instance, *Nicotiana tabacum* exhibited stress effects at 20 mM NH_4_^+^-K^+^ treatment over a 15-day hydroponic period [7]. *Passiflora edulis* showed stress effects above 5.7 mM NH_4_^+^ during a 60-day semi-hydroponic growth [8]. Similarly, *Citrus reticulata* demonstrated stress effects across concentrations (1, 4, and 8 mM NH_4_^+^) during a 6-month semi-hydroponic study [9].

Our study focused on investigating the effects of different ammonium concentrations compared to nitrate nutrition on plant growth, physiology, and metabolite levels in five distinct genotypes. Our analyses revealed a significant genotype effect concerning growth parameters such as biomass, root length, and nitrogen uptake. There was a clear differentiation between the CxLM genotype and the *E. guineensis* genotypes. This finding bears great significance when formulating effective fertilizer management plans, especially during the prenursery stage, as the CxLM genotype demonstrates higher nutritional requirements than the other genotypes.

We ensured that the nitrogen concentrations met the plants’ nitrogen requirements. Over a seven-week exposure period to nitrogen treatments, we observed successful nitrogen uptake and assimilation processes, as indicated by the reduced nitrogen content in the control plants compared to the nitrogen-treated plants. However, prolonged durations or higher nitrogen concentrations could overcome the ammonium tolerance mechanisms in oil palm seedlings, reducing growth. 

One limitation of our experiment should be mentioned. When comparing plants fed with ammonium and nitrate at the same concentration, nitrate-fed plants exhibited higher Ca^2+^ levels in the solution due to the equilibrium of SO_4_^2−^ between the two nitrogen sources with CaSO_4_. Despite this limitation, previous research on Arabidopsis has demonstrated that the extent of ammonium stress remains relatively consistent regardless of variations in calcium availability [10,11,12].

Interestingly, we observed distinct shoot growth patterns between plants fed with nitrate and those fed with ammonium. Nitrate-fed plants displayed greater stability in shoot growth as the concentration increased, while ammonium-fed plants exhibited higher shoot growth. We did not observe a consistent pattern in root fresh and dry weights regarding root growth. However, we noticed a consistent trend in primary root length, with ammonium nutrition generally resulting in longer primary roots than nitrate. These distinct patterns in shoot and root growth lead to a complex behavior regarding the allocation of resources between the root and shoot systems, as reflected in the root-to-shoot ratio influenced by the interaction of nitrogen source, concentration, and genotype. 

There is a tendency for a decrease in the root-to-shoot ratio in ammonium-fed plants compared to nitrate-fed plants as the nitrogen concentration increases. This response aligns with observations in rice, a crop known for its preference for ammonium (NH_4_^+^) [13]. Conversely, crops sensitive to ammonium show an increase in the root-to-shoot ratio [5,14]. 

Regarding photosynthesis the nitrogen source did not have an apparent influence on the net photosynthetic rate. However, there was a slightly significant interaction between the nitrogen source and concentration. Moreover, no other significant differences were found between plants supplied with ammonium and those supplied with nitrate regarding gas exchange and fluorescence parameters.

In contrast, a recent study conducted on blackberry seedlings demonstrated that ammonium nutrition improved overall plant growth, particularly in the roots, and enhanced the net photosynthetic rate [15]. Furthermore, another study on canola revealed varying degrees of resistance to ammonium toxicity, with specific genotypes showing greater shoot and root dry weights in response to high soil ammonium levels [16]. These findings emphasize the complexity of plant responses to different nitrogen sources. 

Regarding metabolites, we observed that as the nitrogen concentration increased, there was also an increase in the content of amino acids. Additionally, we noticed a trend towards increased amino acids and proteins under ammonium nutrition, although this trend was not statistically significant in leaves. This behavior has already been reported, primarily in wheat and sorghum roots [17,18] and rapeseed shoots [19]. Both shoots and roots are essential in ammonium assimilation in oil palm seedlings. The higher levels of amino acids resulting from ammonium nutrition could have advantages, particularly in fruit filling, as amino acids are essential components. However, there could also have disadvantages, such as increased attraction of pests and susceptibility to diseases. In this regard, Marino and Moran [20] reported that ammonium nutrition increased the accumulation of defense-related secondary metabolites, which can help plants resist pathogen attacks. Considering these findings, it would be interesting to investigate in future studies whether the accumulation of amino acids is associated with resistance or sensitivity to bud rot disease, Colombia’s primary oil palm disease. 

The complex interaction of ammonium source, concentration, and genotype influences the levels of reducing sugars in leaves. Similarly, in roots, the levels of reducing sugars depend on the interaction between nitrogen concentration and genotype. On average, there is a slight tendency towards higher root-reducing sugars under ammonium nutrition. Interestingly, the leaves, on average, exhibited higher levels of reducing sugars compared to the roots. This finding aligns with the observations made in the study conducted on blackberry seedlings, which reported higher levels of soluble sugars in leaves compared to roots [15].

We observed that ammonium-fed plants exhibited a higher N uptake in the shoot than the root, resulting in more significant biomass accumulation. Furthermore, as the concentration of ammonium increased, there was a corresponding increase in N uptake in the shoot. In contrast, when examining the root, the N uptake under ammonium nutrition varied depending on the genotype, but on average, it was higher compared to nitrate-fed plants. This observation is consistent with the protective mechanism of roots assimilating ammonium to avoid potential toxicity and has been previously reported in wheat [18]. 

Despite not finding significant differences for most of the physiological parameters evaluated here between ammonium and nitrogen nutrition, this is a positive outcome, considering that ammonium nutrition is generally stressful for plants and its use with nitrification inhibitors is environmentally friendly. Although the concept of nitrogen use efficiency (NUE) is more related to the amount of nitrogen applied that is recovered in the harvest, our assessment of biomass, which is directly related to yield in some crops like cereals, suggests a positive outcome. However, if future projects aim to identify specific metabolites that explain the plant’s performance, we recommend evaluating other metabolites such as antioxidants, organic acids, and enzyme activities related to carbon and nitrogen assimilation. It is crucial to approach ammonium fertilization cautiously, considering the soil’s complexity and dynamic nature, where ammonium is converted to nitrate, especially under aerobic conditions. Therefore, achieving optimal NUE requires matching nitrogen preference and considering other factors such as inhibitors, water irrigation regimens, N fertilizer type, timing, and placement.

## 4. Materials and Methods

### 4.1. Growth Conditions and Experimental Design

Germinated seeds of African oil palm *Elaeis guineensis* (Deli x AVROS (DxA); Deli x La Mé (DxLM); Dura x Pisífera big (DxP big, annual growth rate of 0.6 m year^−1^); Dura x Pisífera small (DxP small, annual growth rate of 0.29 m year^−1^), and one interspecific OxG hybrid (Coari x La Mé (CxLM)) were grown under greenhouse conditions at the experimental station “Palmar de la Sierra” in Zona Bananera, Magdalena (Colombia). Seeds were sown directly in 3 L pots containing a mixture of perlite and vermiculite (1:1; *v*/*v*). They were watered frequently with deionized water. At week seven, the seeds were removed. Nitrogen treatments were initiated by watering initially 50 mL and then 100 mL of nutrient solution every Monday, Wednesday, and Friday with 5, 10, or 15 mM N, corresponding to 0.16, 0.32, or 0.483 g of total nitrogen at the end of the experiment, respectively. Nitrogen was provided as Ca(NO_3_)_2_ or (NH_4_)_2_SO_4_. The nutrient solution contained 0.7 mM CaSO_4_, 1.47 mM KH_2_PO_4_, 0.85 mM MgSO_4_, 2.68 mM KCl, 0.5 mM CaCO_3_, 0.1 mM NaFeEDTA, 16.5 M Na_2_MoO_4_, 3.5 M ZnSO_4_, 16.2 M H_3_BO_3_, 0.47 M MnSO_4_, 0.12 M CuSO_4_, 0.21 M AlCl_3_, 0.126 M NiCl_2_, and 0.06 M KI. To compare the two nitrogen sources at each concentration, plants fed with NO_3_ were provided with CaSO_4_ to equalize the supply of SO_4_^−2^ with the NH_4_^+^ source. The pH of the solution was set at 6.3. A complete randomized block design with five replicates and an experimental unit of three plants was used. Since no prior information was available on the time required for plants to take up and assimilate nitrogen during the seedling stage, we included control treatments without nitrogen (0 N). As soon as we observed symptoms of severe nitrogen deficiency, such as reduced growth and leaf chlorosis, we initiated harvest.

### 4.2. Gas Exchange, Biomass Content, Root Morphology, and Tissue Metabolite Analysis

Gas exchange and fluorescence parameters were measured during week 11 using an Infra-Red Gas Analyzer (IRGA), LI-6800 (LI-COR Inc., Lincoln, NE, USA). The gas exchange component of the instrument was set to a photosynthetic photon flux density (PPFD) of 1000 µmol m^−2^ s^−1^. The airflow rate into the chamber was maintained at 300 µmol s^−1^, while the leaf temperature was kept at 28 °C. Relative humidity was 60%, and the chamber’s CO_2_ concentration was 400 µmol mol^−1^. The fluorometer used rectangular flashes of 10,000 µmol photon m^−2^ s^−1^ intensity and 250 kHz flash modulation rate. Fluorescence measurements were taken in the dark (500 Hz modulation) and light (50 kHz modulation). All measurements were conducted on the first fully expanded leaf. The gas exchange parameters measured included net photosynthetic rate (A_n_), stomatal conductance (g_s_), intercellular CO_2_ (C_i_), and transpiration rate (E). The fluorescence parameters measured included the maximum quantum yield of PSII (F_v_/F_m_), electron transport rate (ETR), the quantum yield of photosystem II (PhiPS2), and non-photochemical quenching (NPQ). Dark-acclimated leaves were measured between 8:00 p.m. and 9:30 p.m., while light-acclimated leaves were measured between 8:30 a.m. and midday.

At week 14, shoots and roots were harvested and weighted. Then, the primary root (PR) and lateral root (LR) lengths were measured using RhizoVision Explorer software, Version 2.0.2 [21]. The roots were classified according to diameter: primary roots had a diameter >1.5 mm and lateral roots had a diameter <1.5 mm. Plant fractions were either oven-dried at 80 °C for 48 h or flash-frozen in liquid nitrogen and stored at −80 °C. Five replicates were collected per condition, and three seedlings were combined in one experimental unit. 

Chlorophylls and amino acids were extracted with ethanolic extraction, while proteins were extracted from the pellet obtained from the ethanolic extraction and measured via colorimetry [22]. Reducing sugars were measured by the Nelson–Somogyi method [23]. The total N was extracted by wet acid digestion and determined using colorimetry. The N uptake value was calculated for each organ using the following formula:

$$N\;\mathrm{uptake}\;(\mathrm{mg}\;N\;{\mathrm{organ}}^{-1})=\mathrm{organ}’s\;N\;\mathrm{content}\;\times \;\mathrm{organ}’s\;\mathrm{dry}\;\mathrm{weight}.$$

### 4.3. Statistical Analysis

The statistical analyses were conducted using the R statistical software Version 4.3.0 (R Core Team, 2023). A multivariate analysis approach was applied to 22 of the measured parameters. The principal component analysis (PCA) from the FactoMineR package and the hierarchical clustering analysis (HCA) from the heatmap2 package were utilized to visualize the data and identify associations among the measured traits, genotypes, nitrogen source, and nitrogen concentrations.

Subsequently, an analysis was performed using a factorial ANOVA to compare various factors, including nitrogen source (N), nitrogen concentration (C), and genotype (G) as well as their interactions (N × C, N × G, C × G, and N × C × G), using the linear model (LM) with type III Sum of Squares (stats-package). To determine significant differences between the nitrogen treatments and the control groups, orthogonal contrasts were specifically established based on genotype, utilizing the multcomp package. Before the analysis, we verified the data’s normality and homogeneity of variance.

## 5. Conclusions

Our study investigated the effects of different ammonium concentrations compared to nitrate nutrition on plant growth, physiology, and metabolite levels in five distinct genotypes. The findings demonstrated a significant genotype effect on growth parameters, highlighting the importance of considering genotype-specific nutritional requirements in fertilizer management plans. Notably, oil palm seedlings have a relatively high tolerance to ammonium, showing equal or more significant growth compared to plants grown with nitrate. Shoot growth was significantly higher in ammonium-fed plants, with the difference increasing at higher nitrogen levels. In terms of root growth, no consistent pattern was observed, with a predominant allocation of photoassimilates to the shoot rather than the root under ammonium nutrition. We did not observe a strong effect of ammonium on physiological parameters but found an increase in amino acid content with higher ammonium concentration, suggesting enhanced nitrogen assimilation. Supporting this notion, ammonium-fed plants displayed higher nitrogen uptake in both the shoot and root. Our findings indicate that ammonium nutrition could be an environmentally friendly approach for oil palm cultivation. 

## Data Availability

Not applicable.
